# Collapse of fragile Chinese Swamp Cypress forest

**DOI:** 10.1126/sciadv.adt1736

**Published:** 2025-04-23

**Authors:** Ning Wang, Ping Ding, Xingfang Ding, Yongqiang Zong, Weidong Sun

**Affiliations:** ^1^ State Key Laboratory of Deep Earth Processes and Resources, Guangzhou Institute of Geochemistry, Chinese Academy of Sciences, Guangzhou 510640, China.; ^2^ State Key Laboratory of Nuclear Physics and Technology, Peking University, Beijing 100871, China.; ^3^ Key Lab of Guangdong for Utilization of Remote Sensing and Geographical Information System, Guangdong Open Laboratory of Geospatial Information Technology and Application, Guangzhou Institute of Geography, Guangdong Academy of Sciences, Guangzhou 510070, China.; ^4^ Key Laboratory of Ocean Observation and Forecasting, Institute of Oceanology, Chinese Academy of Sciences, Qingdao 266071, China.; ^5^ Ocean Sciences and Interdisciplinary Frontiers, Laoshan Laboratory, Qingdao 266237, China.; ^6^ University of Chinese Academy of Sciences, Beijing 100049, China.

## Abstract

The tertiary relict species *Glyptostrobus pensilis*, formerly widespread in the Pearl River Delta, experienced a sudden decline and was on the brink of extinction in the late Holocene. The mechanism behind is still in debate. Here, using palynological records and principal components analysis, we show that *Glyptostrobus pensilis* is sensitive to anthropogenic disturbance. Elaborate ^14^C results reveal that the forests ended around 2.1 thousand years before the present, with mild contemporaneous climate changes. The presence of burned marks on buried standing stumps suggests that the forests were destroyed by fire, consistent with fire attacks by the Han army during the conquest of the Nanyue Realm in 111 BCE. The swamp preserved the stumps underwater from the fire. Meanwhile, the increases in Poaceae and pioneer plants indicate a boost of human activity after the two conquests during 221 to 111 BCE, as supported by the increases in anthropogenic metal concentrations and population records.

## Teaser

Fire attacks during the war and increase in human activity confirmed by “buried forest” of Swamp Cypress in the Pearl River Delta.

## INTRODUCTION

**Fig. 1.**
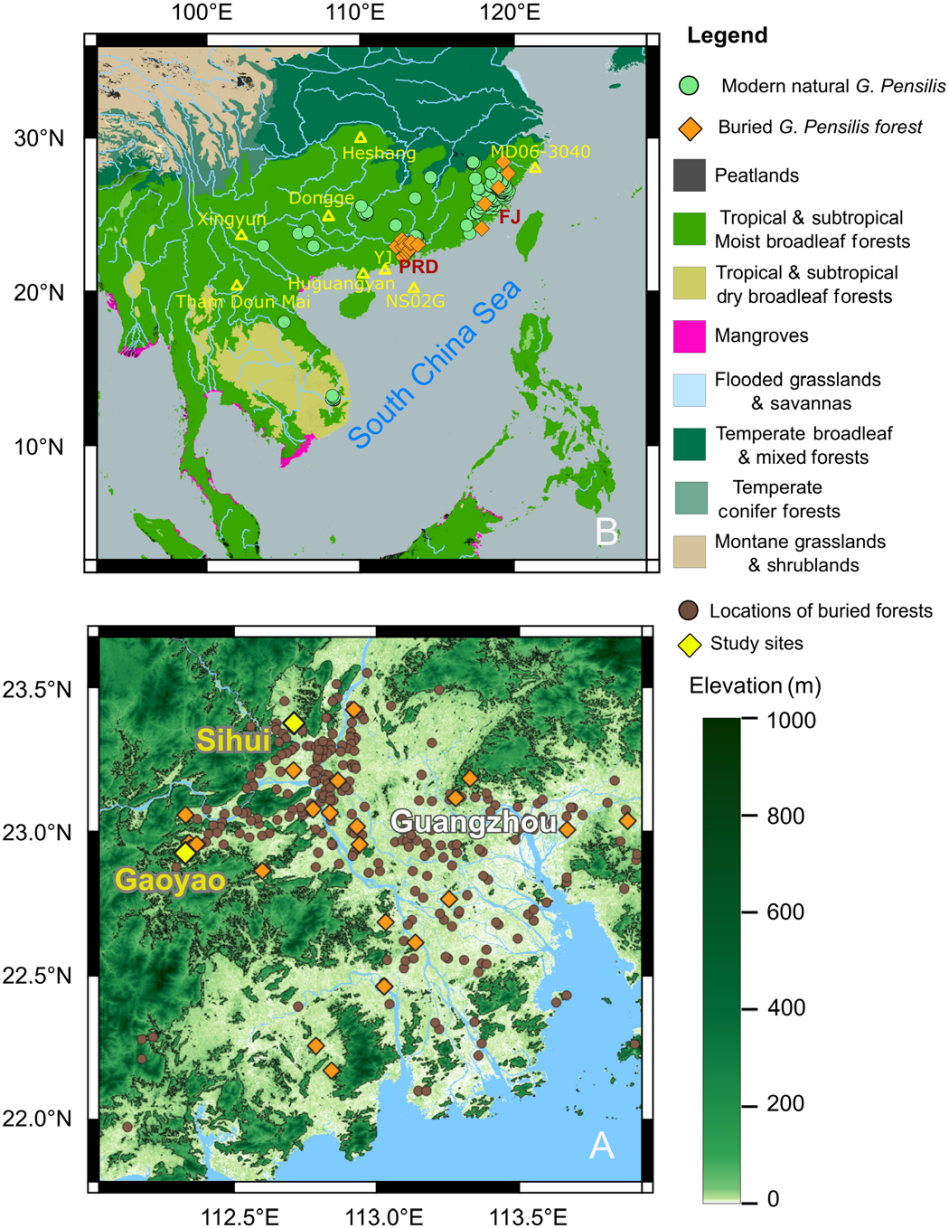
Distribution of extant and buried *G. pensilis* in Asia and the PRD. (**A**) Distribution of buried forests (brown circles) and those identified as ancient *G. pensilis* forests (orange diamonds) in the PRD. Yellow diamonds indicated the study sites of Gaoyao and Sihui (*14*, *71*). (**B**) Distribution of extant and buried *G. pensilis* within ecoregions based on Ecogions2017. Yellow triangles indicate the locations of paleoclimate records mentioned in the text.

War is doubtlessly one of the most critical anthropogenic drivers of modern land cover changes (*1*–*3*). However, the impact of war on land cover in history has often been ignored or underestimated. One of the few historical records documenting notable vegetation destruction due to war is Socrates’ witness of the Spartans’ devastation of farms and fields and the chopping down olive orchards and vineyards during the Peloponnesian War in the fifth century BCE (*4*). Over the past three decades, a series of studies have uncovered massive peat layers widespread in valley lowlands, riparian corridors, and river floodplains at the head of Pearl River Delta (PRD), indicating formerly widespread of swamp forests in the PRD (*5*–*7*). This peat-layer complex, with a thickness of 2 to 6 m and covering an area of over 2000 km^2^ (Fig. 1A), dates to 2 to 6 thousand years ago (ka) (*6*–*8*). The organic matter in peat layers is well preserved, resulting in a total organic carbon (TOC) content as high as 40 to 60%. These peat layers are locally known as “buried ancient forest,” because many buried trees appear fresh and most stumps are found still standing (fig. S1, B, C, and E) (*6*, *7*, *9*). The land cover in the PRD region, originally dense forests, has now been changed into farmlands.

One of the most common tree species in these peat layers is *Glyptostrobus pensilis*, also known as the Chinese Swamp Cypress, a typical Tertiary relict flora, indicating that extensive peat swamp forests existed in the PRD (*10*). *G. pensilis*, once widespread in the Northern Hemisphere from the Cretaceous to the early Pleistocene, is now a critically endangered species on the Red List (*11*). It is heliophilous and tolerates waterlogging, generally growing in riparian lands, river deltas, lowlands, sometimes on flooded or waterlogged soil, and along rice fields in the tropics and subtropics (*12*, *13*). Its suitable habitats have an optimal annual temperature range of 12° to 25°C and mean annual precipitation between 1200 and 2230 mm (table S1) (*8*, *12*). The distribution range of *G. pensilis* (Fig. 1B) was previously underestimated according to “Investigation Ancient and Big Trees” (*14*). Investigation results show that most areas of southeastern China are suitable habitats for *G. pensilis* forests (*14*). However, the extant native *G. pensilis* only survives in remote inland areas where human activities have not destroyed forests for agriculture purposes and have become extremely rare in the coastal area, where the human population is dense (*15*). Most *G. pensilis* populations are small and scattered, unable to provide the ecosystem services they once did.

The cause of the collapse of the *G. pensilis* forest is in debate. Early research attributed it to climatic factors, such as cooling events (*7*), droughts (*6*, *9*, *16*), and inundation by seawater or freshwater (*17*–*19*). However, recent studies consider human activities, primarily the development of agriculture, as the cause (*8*). As the historical political center of the Nanyue Realm (present-day Guangdong and Guangxi in China and the northern part of Vietnam), the PRD region was the major hotspot for battles and garrisons during conflicts between the local regime and the central government. During these periods, the land cover experienced substantial changes. The Nanyue Realm was established in 204 BCE after the conquest of regional tributes by the Qin Empire from 221 to 214 BCE, by a former general of the Qin Empire (*20*). Less than one century later, in 111 BCE, the Nanyue Realm was defeated by the Han Empire (*20*, *21*). It is well documented that the Han army launched fire attacks during the war that led to the defeat of the Nanyue Realm. However, the extent to which these wars altered the local land cover remains unclear. Here, we unburied the succession history of the buried ancient forest by examining sediments from Gaoyao and Sihui in the PRD using chronological, palynological, and sedimentological methods. The results indicate that irreversible changes in land cover were likely the result of wars and subsequent population growth and introduction of advanced agricultural technologies. This study specifically focuses on the interaction among climate events, human society, and regional land cover over the past 5000 years.

## RESULTS

### Sedimentary sequences and chronology

The buried forests in the PRD are noteworthy. In Gaoyao, peat layers rich in stumps and root remains are c. 5- to 6-m thick (fig. S1A), occupying an area of about 3 ha. The peat layer in Sihui is more than 3-m thick and spans about 2 ha (fig. S1E). The stumps in these peat layers are well preserved, with the largest ones nearly reaching 2 m in diameter (fig. S1E). Many buried stumps in the upper peat layer remain standing, and some have burn marks on their tops (fig. S1, A, C, and E). We also find charcoal in the top clay layer that overlies the peat layers in Gaoyao. Besides, the profiles in Gaoyao have two distinct white clay interlayers (fig. S1, A and B). The profile in Sihui has a thin gray clay layer and a thick yellow cultivated soil layer that overlies the peat layers. Unlike Gaoyao, this profile does not have the two white clay layers typically found within the peat layer, which may indicate potential sedimentary hiatuses or disturbance (fig. S1, D and E, and Materials and Methods).

Dating results indicate that the base of the peat layers in Gaoyao dates to 4915 ± 70 years (a) before the present (B.P.) and that in Sihui to 5149 ± 77 a B.P. (tables S2 and S3 and figs. S2 and S3). Profile SHS1 shows a clear sedimentary hiatus from 3250 ± 58 to 1073 ± 59 a B.P. and possibly another during 4852 ± 31 to 4190 ± 56 a B.P., based on a low sedimentary rate. Chronological results indicate that profile GYS1 provides more continuous and detailed records for vegetation and climate change. Therefore, we focus primarily on the results from GYS1. The dating of the stumps indicates the onset (innermost part) and termination (outmost part) of tree growth. We have compiled previous stump dating results with ours (table S4), which have an average age of 2040 a B.P., providing insights into the collapse of the swamp forest in the PRD region.

### Land cover change in the PRD region

**Fig. 2.**
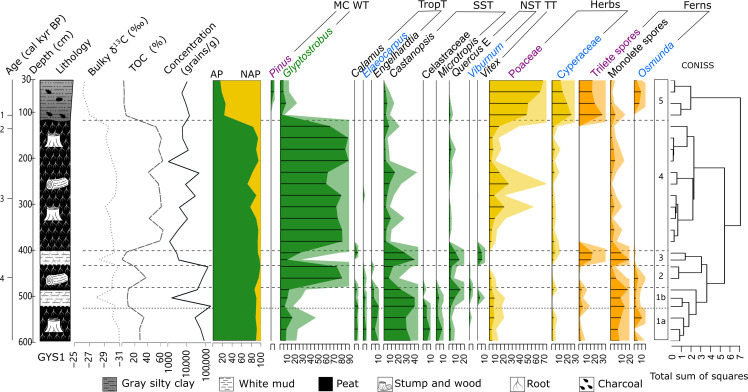
Sedimentary, organic carbon, and selected palynological records from GYS1. The selected taxa are categorized into montane conifers (MC), wetland trees (WT), tropical trees (TropT), south subtropical trees (SST), north subtropical trees (NST), temperate trees (TT), herbs, and ferns (for further details, refer to fig. S5 for main palynological taxa records and table S5). Trees, herbs, and ferns are highlighted in green, yellow and orange, respectively. AP denotes arboreal pollen, while NAP denotes non-AP. Dashed lines denote the assemblage zones identified by CONISS (constrained incremental sum of squares).

In profile GYS1, a total of 84 species of pollen taxa were identified, and the vegetation changes have been reconstructed since approximately 4.9 ka (Fig. 2 and fig. S5). Forty-three main palynological taxa (fig. S5), with a total percentage greater than 5%, are listed in table S5 along with their ecological groups. Constrained cluster analysis revealed five pollen assemblage zones. Zone 1 was further divided into two subzones, zone 1a and zone 1b, based on notably different sedimentary characteristics, TOC and δ^13^C values (Fig. 2 and fig. S1A).

#### Zone 1a (4900 to 4300 a B.P.; 596 to 521 cm)

High arboreal pollen (AP; 90 to 92%) percentages indicate a nonwetland forest dominated by the south subtropical tree *Castanopsis* (29 to 35%), with other south subtropical and tropical trees (*Engelhardtia*, *Elaeocarpus*, *Calamus*, and Celastraceae). The forest is dense based on the high AP percentages, high pollen concentration, and high percentages of monolete spores (10 to 14%), which is a characteristic of damp, shady habitats. High TOC levels (28.07 to 34.61%) and more negative δ^13^C values [between −29.97 and −30.87 per mil (‰)] support the presence of arboreal vegetation (*9*). This phase exhibits a relatively high capacity for carbon fixation and storage, as evidenced by the peat layers.

#### Zone 1b (4300 to 4100 a B.P.; 521 to 485 cm)

Pollen assemblages continue to be dominated by *Castanopsis* (22 to 40%). The increasing percentages of north subtropical (NST) and temperate trees (TT), specifically evergreen *Quercus* (*Quercus* E) and *Vitex*, which together constitute 3 to 16%, suggest a cooling environment. Lower pollen concentration, reduced TOC levels (8.84 to 10.54%), and a positive shift in δ^13^C values (between −27.8 and −30.3‰) indicate a decrease in tree densities, suggesting forest degradation and a relatively high concentration of C4 plants. This phase has poor carbon fixation and storage, as indicated by the white clay layers.

#### Zone 2 (4100 to 3600 a B.P.; 485 to 435 cm)

Pollen assemblages reveal that the vegetation is dominated by a peat swamp forest of *G. pensilis* (74 to 81%), suggesting a warm and/or wet environment. The simultaneous decreases in *Castanopsis* (7 to 12%), *Calamu*s, and *Quercus* E indicate the recession of nonwetland forests, likely due to a high water table level rather than a warm climate. High pollen concentration, elevated TOC (20.34 to 38.26%), and more negative δ^13^C values (between −29.97 and −30.72‰) suggest a dense swamp forest. The dark peat and well-preserved trees suggest substantial carbon fixation and carbon storage in this phase probably due to a high water table level.

#### Zone 3 (3600 to 3400 a B.P.; 435 to 405 cm)

Pollen assemblages show the degradation of peat swamp forest, as evidenced by low percentages of *G. pensilis* (9 to 26%). Decreased pollen concentration and reduced TOC levels (1.00 to 7.51%) suggest a reduction in tree densities. The climate appears to have become colder, as indicated by higher percentages of *Quercus* E (9 to 14%) and *Vitex* (~6%). In addition, high percentages of trilete spores (mostly *Dicranopteris* type, 16 to 18%) and monolete spores (14 to 25%) suggest a dry environment. This white clay layer shows poor carbon fixation and storage conditions.

#### Zone 4 (3400 to 1900 a B.P.; 405 to 130 cm)

The vegetation is once again dominated by the peat swamp forest of *G. pensilis* (60 to 89%) with sporadic nonwetland forest (featuring *Castanopsis* at 2 to 15% and monolete spores at 1 to 8%) surrounding it. This distribution is attributed to warm and wet climate conditions. Negative δ^13^C values with small fluctuations (between −29.0 and −30.5‰) suggest a stable swamp forest during this period. Occasionally high percentages of Poaceae (sometimes up to 20 to 25%) indicate early human activities in the PRD. Relatively high pollen concentration, high TOC (41.91 to 63.60%), and a peat layer with well-preserved stumps together indicate high carbon fixation and substantial carbon preservation in this phase. Many stumps in this layer remain standing and have burned marks on their tops.

#### Zone 5 (1900 to 0 a B.P.; 130 to 0 cm)

The dominant vegetation transitions from peat swamp forests to anthropogenic grasslands and/or croplands, as indicated by low AP percentage (16 to 27%) and relatively positive δ^13^C values (between −26.1 and −27.0‰). The herbs are dominated by Poaceae (49 to 71%), which is an index of rice cultivation when concentrations exceed 36% (*22*). High percentages of pioneer species, such as *Pinus* (up to 4%) and trilete spores (up to 30%), also suggest frequent anthropogenic disturbance in the PRD. High percentages of Cyperaceae (11 to 26%), wet-loving and sub-aquatic grasses, and *Osmunda* (0 to 9%) indicate a persistently moist environment. However, low TOC levels (3.30 to 7.03%) and the presence of silty clay imply poor carbon fixation and storage during this phase. This layer contains a large amount of charcoal, suggesting substantial human activity and land-use changes.

### Principal components analysis of pollen results

**Fig. 3.**
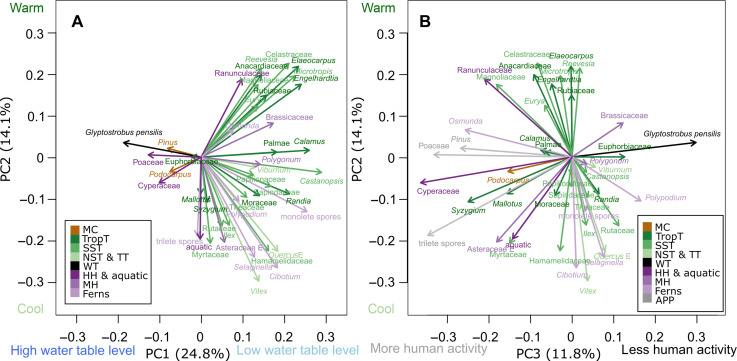
PCA ordination diagram of main palynological taxa from GYS1. (**A**) PC1 versus PC2. (**B**) PC3 versus PC2. Different colors denote MC, TropT, SST, NST trees, TT, WT, hygrophyte herbs (HH), mesophyte herbs (MH), anthropogenic and pioneer plants (APP), and ferns.

Principal components analysis (PCA) results highlight the impacts of natural and anthropogenic drivers on the variation of different taxa. PC1 (PCA axis 1) features most wetland trees (*G. pensilis*), hygrophyte herbs, and montane conifers in its negative phase (Fig. 3A), likely indicating a water-logged environment with a high water table level in the lowlands between hills. Most arboreal plants, intolerant of prolonged waterlogging, are found in the positive phase of PC1. Thus, PC1 could serve as a proxy for the water table level, with more negative values indicating a more waterlogged environment due to a higher water table level. PC2 appears to be associated with temperature (Fig. 3A), as most NST and TT are in the negative phase, while most tropical trees are in the positive phase. Consequently, high PC2 values may indicate high temperatures.

PC3 is a robust proxy for anthropogenic disturbance, and *G. pensilis* is highly sensitive to human activities (Fig. 3B). PCA results indicate that Poaceae, Cyperaceae, *Pinus*, and trilete spores fall in the negative phase of PC3. Poaceae commonly serves as an indicator of human disturbance or agricultural activity. As wetland aquatic herbs, Cyperaceae abundance increases with the rise of rice cultivation, as paddy fields provide suitable habitats. Trilete spores, most of which are *Dicranopteris* type, thrive in areas burned repeatedly and are closely associated with land clearance for agriculture (*23*). *Pinus* species are typical pioneer plants, indicative of secondary forest after severe fires or deforestation. Therefore, low PC3 values suggest substantial anthropogenic disturbance. The high PC3 score for *G. pensilis* suggests that it is more sensitive to human activity than other plants, consistent with the observation that most extant native *G. pensilis* population are found in remote areas, far from human influence.

**Fig. 4.**
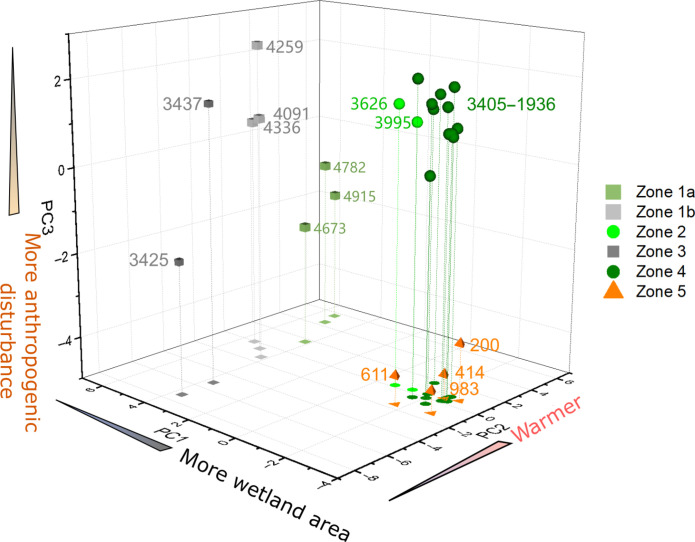
3D scatterplot visualization of PC1, PC2, and PC3 for samples from different zones. The number adjacent to each sample denotes its modeled mean age (a B.P.). Dotted lines link the 3D scatterplots to their corresponding projections on the basal plane.

On the basis of PC1, PC2, and PC3 values, the three-dimensional (3D) scatter of sedimentary samples (Fig. 4) illustrates variations in water table level, temperature, and anthropogenic disturbance in the PRD region. The environment was warm from 4.9 to 4.6 ka B.P., followed by a cold period of approximately 300 years starting around 4.3 ka. Subsequently, moisture increased at 4.0 ka, coinciding with the prosperity of *G. pensilis* forest. Around 3.5 ka, the environment turned cold and dry for nearly 100 years, before returning to warm and wet conditions for approximately 1500 years. *G. pensilis* forest was thriving again during this warm and wet period. This suggests that changes driven by natural factors are reversible. However, a marked shift in land cover occurred around 2.1 ka, likely due to anthropogenic disturbance, as evidenced by the abrupt decrease in PC3 values. This disturbance was so intense that *G. pensilis* has not been the dominant plant over the past 2000 years, despite environmental conditions remaining suitable for its growth, as suggested by similar PC1 and PC2 values.

## DISCUSSION

### Impact of climate change on land cover in the PRD

**Fig. 5.**
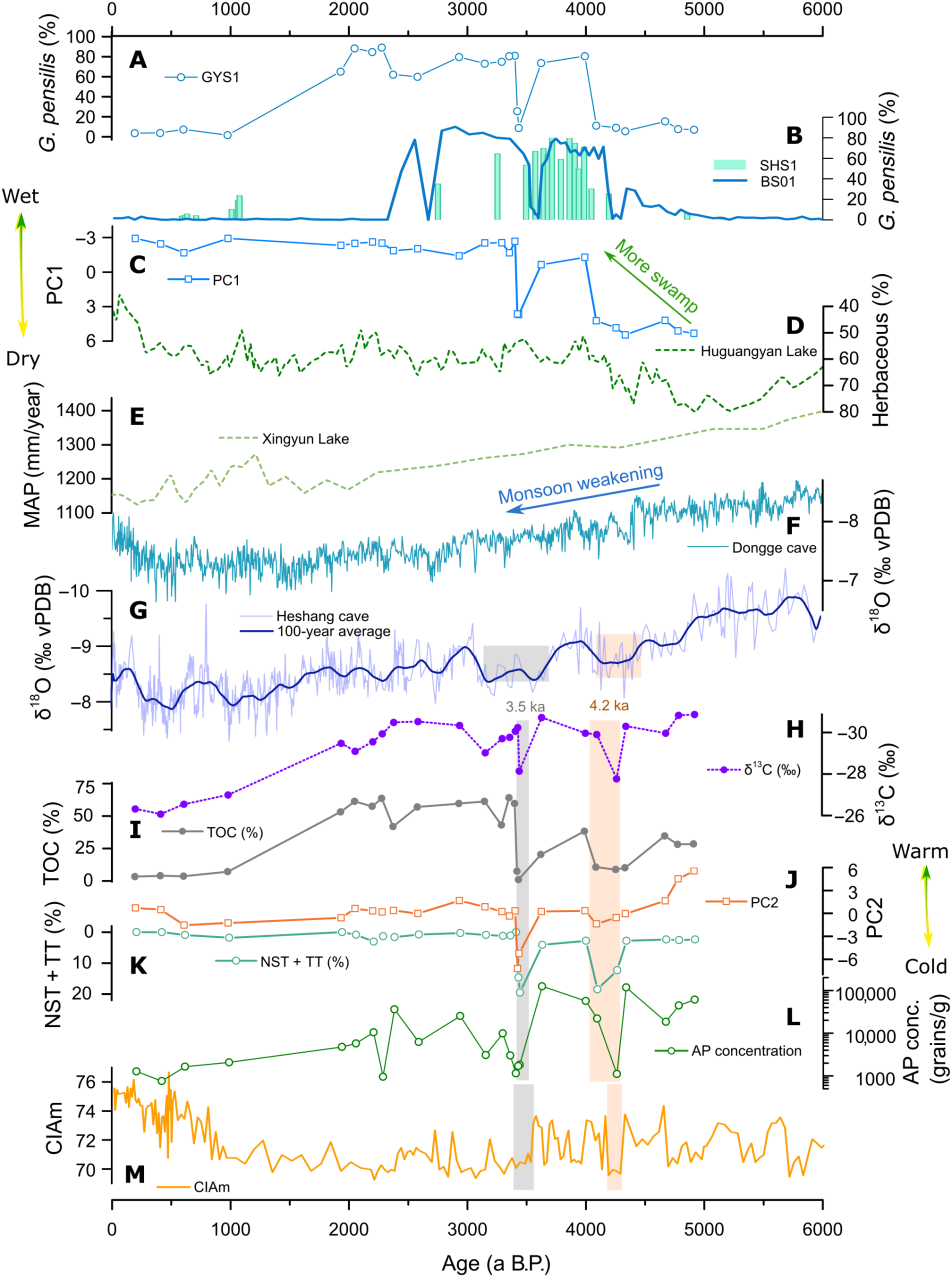
Climate change in the PRD region as reconstructed by multiple records from GYS1 and comparison with moisture records from the coastal area and inland areas. (**A**) Percentage records of *G. pensilis* from GYS1 in this study. (**B**) Percentage records of *G. pensilis* from SHS1 in this study and BS01 from Gaoyao in another study (*19*). (**C**) PC1 value records from GYS1. (**D**) Herbaceous percentage records in sediments from Huguangyan Marr Lake in the coastal area (*25*). (**E**) Pollen-derived mean annual precipitation (MAP) reconstruction from Xingyun Lake (*27*). (**F**) Stalagmite δ^18^O records from Dongge cave (*26*). (**G**) Stalagmite δ^18^O records and the 100-year average curve from Heshang cave (*28*). (**H** to **L**) Multiple palynological proxies and organic carbon records in GYS1, including (H) δ^13^C values, (I) TOC contents, (J) PC2 values, (K) pollen percentages of NST and TT, and (L) AP concentrations. (**M**) Chemical weathering proxy CIAm records in YJ cores from the northern South China Sea (*56*).

In the PRD region, a notable change in the land cover is the replacement of nonwetland forests with extensive swamp forests around 4 ka, a shift that is associated with changes in the water table level (Fig. 5, A to C) (*19*). Variations in PC1 values (Fig. 5C), as a proxy for the water table level, indicate that moisture levels in the PRD region stabilized after an increase of around 4 ka. The water table level is influenced by both the sea level and precipitation. The sea level in the PRD reached its maximum around 7 ka B.P. and has changed minimally over the past 7000 years (*24*). Therefore, precipitation is the primary influence on the water table level. The herbaceous percentage record from Huguangyan Marr Lake in the western coastal area of Guangdong (Fig. 5D) also indicates that precipitation was relatively stable following an increase during 4.0 to 4.5 ka B.P. (*25*). Precipitation variations in the coastal areas of Guangdong differ from those in the inland areas. Precipitation reconstructions in Xingyun, Dongge, and Heshang exhibit a slight decrease over the past 6000 years (Fig. 5, E to G) (*26*–*28*). Many climate reconstructions indicate a general decrease in precipitation and moisture levels following the Holocene Climatic Optimum to the north of Guangdong (*29*, *30*). This decrease was caused by the weakening of the Asian Summer Monsoon (*26*, *31*), which led to the southward movement of the rain belt in South China, resulting in increased precipitation of around 4 to 4.5 ka in the coastal areas of Guangdong.

Another feature of land cover change is the abrupt shifts in vegetation succession, triggered by two crucial climate events, as evidenced by multiple palynological proxies and organic carbon records (Fig. 5, A, C, and H to L). The first event occurred between 4336 ± 59 a B.P. and 4091 ± 55 a B.P., reflecting the impact of the global 4.2-ka event on the PRD region. The 4.2-ka event is a pronounced climate extreme, which is associated with abrupt global droughts and/or cooling events that contributed to the rise and fall of several cultures (*32*–*35*). The 3D scatterplots of sedimentary samples (Fig. 4) show that the main change occurred in PC2, a proxy for temperature. The low percentages of NST and TT (Fig. 5K) suggest a cooling event in the PRD region during 4.3 to 4.1 ka B.P. The decreased temperature caused an overall decline in various plant species, as evidenced by a low TOC level and AP concentration (Fig. 5, I and L). The cold condition also reduced the chemical weathering rate, which is supported by the decline in the CIAm record, a weathering proxy, from an ocean sediment core near the PRD region (Fig. 5M).

### The 3.5-ka event in the PRD and ITCZ

**Fig. 6.**
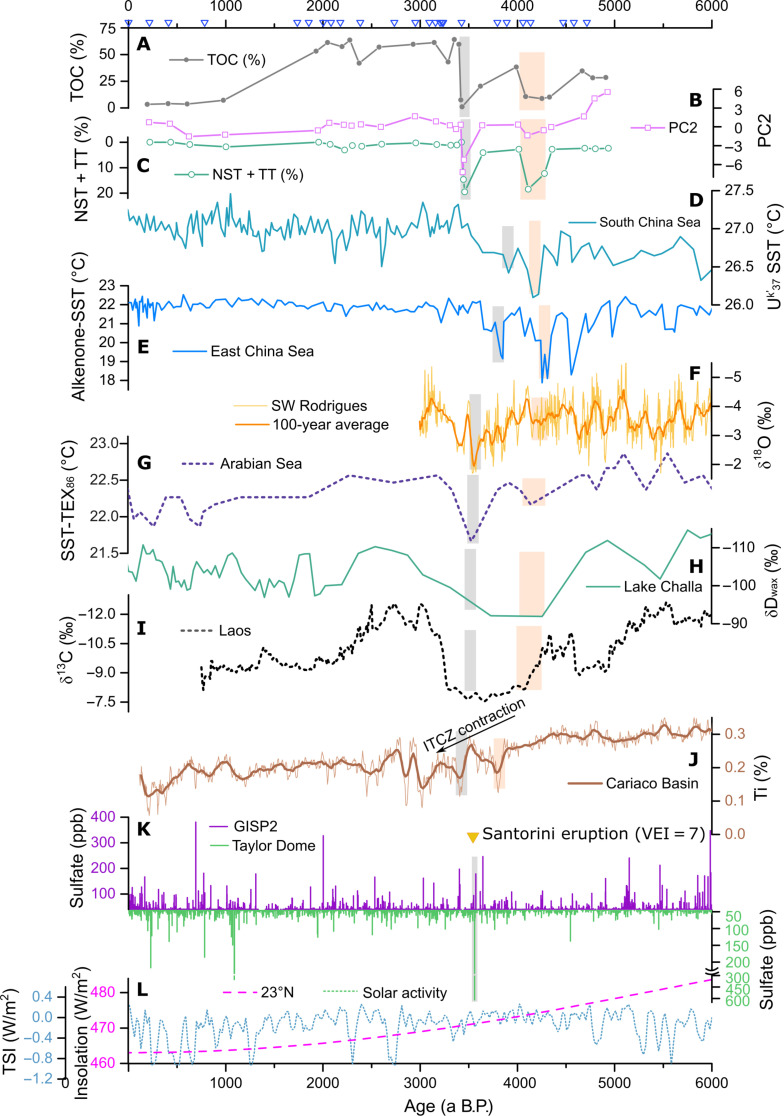
Comparison with records from the ITCZ. (**A** to **C**) TOC content, PC2 value, and pollen percentage records of NST and TT from GYS1. (**D**) Sea surface temperature proxy of U^k^′_37_ records from NS02G in the northern South China Sea (*39*). (**E**) Alkenone records of sea surface temperature from MD06-3040 in the East China Sea (*40*). (**F**) Stalagmite δ^18^O records from La Vierge cave in southwest Rodrigues (*41*). (**G**) Sea surface temperature TEX86 records from ocean sediments in the Arabian Sea (*42*). (**H**) δD of the wax in sediments from Lake Challa in East Africa (*44*). (**I**) stalagmite δ^13^C records from Tham Doun Mai cave in northern Laos (*43*). (**J**) Ti percentage in sediments from the Cariaco Basin (*45*). (**K**) Sulfate content in ice cores from Greenland Ice Sheet Project 2 (GISP2) in Greenland (*50*) and Taylor Dome in Antarctica (*51*). (**L**) Solar insolation at 23°N (*47*) and solar activity (*48*). Gray bars indicate the 3.5-ka event, and orange bars indicate the 4.2-ka event. The locations of some comparison records are marked in Fig. 1B. Blue triangles on the top denote the location for radiocarbon dating. ppb, parts per billion.

The second forest degradation occurred between 3626 ± 61 and 3425 ± 15 a B.P., likely resulted from a cooling and drying event. A high water table level creates a suitable sedimentary environment for preserving organic matters, leading to high TOC levels. So, high PC1 values, positive shifts in δ^13^C values, and low TOC levels (Fig. 5, C, H, and I) all indicate a drought event, which also caused the reduction in *G. pensilis* percentages (Fig. 5A). The positive shift in PC2 values and the percentages of NST and TT (Fig. 5, J and K) suggest a cooling environment. This drought and cooling event is not widely discernible in global paleoclimatic reconstructions, especially at high latitudes (*32*, *36*–*38*). However, many reconstructions from areas influenced by the Intertropical Convergence Zone (ITCZ) show multiple events during 4.3 to 3.4 ka B.P. or a long-term event spanning from 4.3 to 3.4 ka B.P. (Fig. 6, D to J), potentially related to the 3.5-ka event. Sea surface temperature reconstructions (Fig. 6, D and E) reveal multiple cooling events between 4.6 and 3.5 ka B.P. in both the South China Sea and the East China Sea (*39*, *40*). The positive shift in the stalagmite δ^18^O (Fig. 6F) and δ^13^C records from southwest Rodrigues (19°42′S, 63°24′E) indicate mega-drought events around 3.5 ka B.P., which is more notable than the 4.2-ka event (*41*). The sea surface temperature reconstructed by tetraether index of 86 carbon atoms (TEX86) from the Arabian Sea (Fig. 6G) reveals a notable decline associated with the 3.5-ka event, which was even colder than the 4.2-ka event (*42*). Both the stalagmite δ^13^C from northern Laos and the δD value of wax in sediments from Lake Challa in the East Africa (Fig. 6, H and I) show a long-term drought between 4.3 and 3.4 ka (*43*, *44*). The 3.5-ka event is therefore not just a local event driven by the Asian Monsoon but more likely a crucial regional event. According to the ITCZ proxy of Ti percentage in ocean sediments from the Cariaco Basin (Fig. 6J), this event is associated with sudden ITCZ contractions around 3.5 ka B.P. (*45*).

The 3.5-ka event may have been triggered by volcanic activity, i.e., the Santorini eruption at c. 3550 to 3577 a B.P. (*46*). Solar irradiance is ruled out as a driver for the 3.5-ka event, as neither solar insolation nor solar activity variations (Fig. 6L) show strong abnormal fluctuations of around 3.5 ka B.P. (*47*, *48*). Santorini (also known as Thera), the southernmost island of Cyclades in the Aegean Sea, had a volcanic explosivity index (VEI) = 7 eruption during the Late Bronze Age, which was probably the largest volcanic eruption in recorded human history (*49*). This eruption is recognized to have worldwide effects on climate disturbance (*46*). Although this eruption occurred in the North Hemisphere, the sulfate records from the Taylor Dome in Antarctica show a stronger signal than those from Greenland Ice Sheet Project 2 in Greenland (Fig. 6K) (*50*, *51*). This suggests that most volcanic ash was likely to spread to low latitudes and the Southern hemisphere regions with large areas of ocean, thereby inducing a cooling effect on the oceans and subsequently altering ocean circulation patterns (*52*). The cooling of tropical oceans could lead to the ITCZ contraction, which reduced the precipitation and temperature in the PRD region. This disturbance may have persisted for several decades, leaving a discernible signature in some paleoclimate records associated with the ITCZ (*41*, *42*, *53*, *54*). Further, high-resolution records with robust chronology are required for confirming the link between the 3.5-ka event and the Santorini eruption.

### Cause of *G. pensilis* forest collapse in PRD

**Fig. 7.**
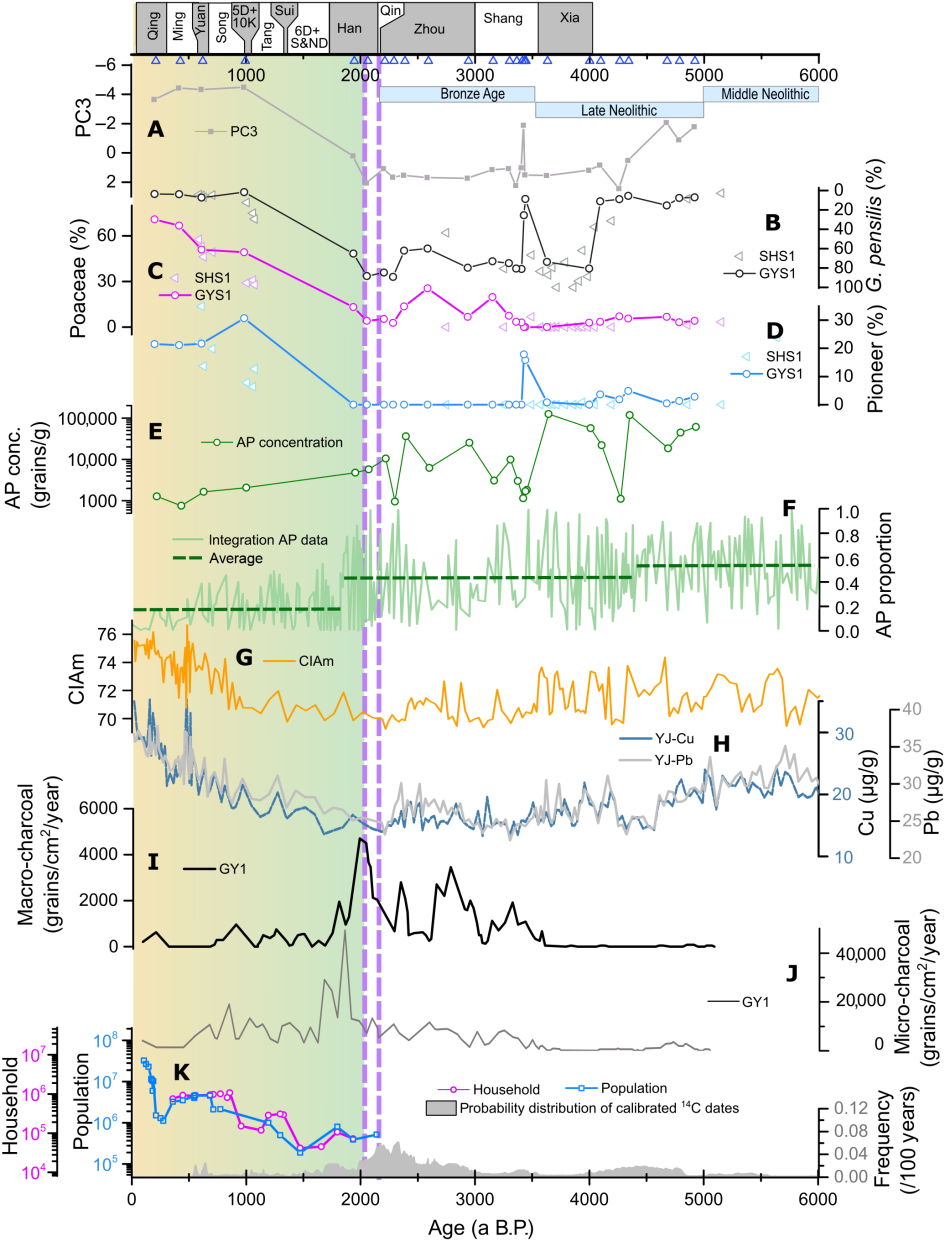
Anthropogenic disturbance reconstruction by GYS1 and SHS1 with comparison to other records in the PRD region. (**A**) PC3 values from GYS1. (**B** to **D**) Pollen percentages of *G. pensilis*, Poaceae, and pioneer plants from GYS1 and SHS1. (**E**) AP concentrations from GYS1. (**F**) AP proportion by the integration data from the cores in the coastal areas of South China (*57*) with dashed lines indicating the average values for different periods. (**G**) Chemical weathering proxy CIAm records in YJ cores from the northern South China Sea (*56*). (**H**) Cu and Pb concentration in YJ cores (*56*). (**I** and **J**) Macro-charcoal and micro-charcoal records in GY1 from Gaoyao (*60*). (**K**) Population and household records in Guangdong and Guangxi (*62*) and the probability distribution of calibrated ^14^C dates in archaeological database in Guangdong and Guangxi (*61*). Purple dashed lines denote the two conquests by Qin Empire during 221–214 B.C and Han Empire during 112–111 BCE.

The collapse of the *G. pensilis* forest occurred approximately 2.1 ka B.P., the cause of which was unlikely to be climate change. Climate change over the past 3000 years was weaker than the two events (the 4.2- and 3.5-ka events). The precipitation proxy of PC1 (Fig. 5C) and the herbaceous percentage in Huguangyan Lake (Fig. 5D) (*25*) did not change much over the past 3000 years. Similarly, the temperature proxy PC2 (Fig. 6B) and sea surface temperature reconstruction in the northern South China Sea (Fig. 6D) (*39*) have been relatively stable since 3.0 ka B.P. Furthermore, the ITCZ position, solar irradiance, and volcanic activity (Fig. 6, J to L) have no abrupt changes around 2.1 ka B.P. In addition, although our results reveal notable land cover changes during the two events (the 4.2- and 3.5-ka events), the *G. pensilis* forest recovered from these climate disturbances (Fig. 7B). Therefore, the collapse of the *G. pensili*s forest around 2.1 ka B.P. cannot be attributed to natural causes. The mild climate change infers that human activity overwhelmed climate factors and became the primary cause for the abrupt collapse of the *G. pensilis* forest around 2.1 ka.

Anthropogenic disturbance began to increase substantially since 2.1 ka B.P. in the PRD region, coinciding with a decline in the percentage of *G. pensilis*. The decrease in PC3 (Fig. 7A), as a proxy for anthropogenic disturbance, indicates an increase in human activity since 2.1 ka B.P. Poaceae, which contains a lot of cereal types such as rice, wheat, and barley, is an indicator of rice cultivation when its concentration is more than 36% (*22*). The concentrations of Poaceae in the top gray clay layer were higher than 49% (Fig. 7C), suggesting the development of agricultural cultivation. Similarly, pioneer plants (Fig. 7D), which often appear after anthropogenic deforestation (such as slash-and-burn agriculture), have also increased over the past 2000 years. Besides, ocean sediment reconstructions show similar increases in Cu and Pb concentrations since 2.1 ka B.P. (Fig. 7H) (*55*). Copper (Cu) is a typical anthropogenic metal pollutant associated with tool manufacturing for rice cultivation and the use of copper cash in ancient China, while lead (Pb) is one of the major components in many living goods from cosmetics to metalware and coins. The increase in Cu and Pb concentrations further supports the boost of human activity in the PRD region since 2.1 ka B.P. In addition, the CIAm variation (Fig. 7G) indicates an increased input of weathered materials due to intensive agriculture at the same time (*56*). Pollen integration data from the cores in the coastal areas of South China also indicate the intensive deforestation after 2.1 ka B.P. (Fig. 7F) (*57*).

However, the collapse of the *G. pensilis* forest cannot be simply attributed to the development of agricultural cultivation. Before the Qin conquest, local populations in South China practiced various forms of slash-and-burn, shifting cultivation, along with hunting and gathering (*58*). Rice cultivation expanded to the far south of China during 4.4 to 3.5 ka B.P. (*59*). In the PRD region, the increase in macro-charcoal since 3.7 ka B.P. (Fig. 7I) suggests frequent local slash-and-burn cultivation activities (*60*). Similarly, the probability distribution of calibrated ^14^C dates from Neolithic and Bronze Age archaeology (Fig. 7K) indicates a population increase since 3.5 ka B.P. (*61*, *62*). However, this increase was gradual, and its impact was insufficient to cause substantial land cover changes in the PRD region during 3.5 to 2.1 ka B.P. The high AP concentration and AP proportion between 3.5 and 2.1 ka B.P. (Fig. 7, E and F) indicate that forests still dominated the vegetation. Although occasional increases in Poaceae percentages (Fig. 7C) indicate agricultural activities, their impact on local land cover was limited, as evidenced by the extremely low percentages of pioneer plants and the high percentages of *G. pensilis* (Fig. 7, B and D). Instead, a more marked event rather than agricultural cultivation explosively altered the peat swamp forest, pushing it past the point of no return.

The transition from natural driver dominance to anthropogenic driver dominance in vegetation change in the PRD region occurred around 2.3 to 2.0 ka, probably due to two critical historical events. The first was the conquest by the Qin Empire from 221 to 214 BCE, which included three expeditions (*20*). In 221 BCE, the First Emperor of Qin sent an army of 500,000 soldiers led by Tu Sui (*20*, *63*). Later, a large population of fugitives from justice, indentured sons-in-law, merchants, exiled men, and injustice law officers were sent to reinforce the garrison in South China between 214 and 213 BCE (*64*). Furthermore, 15,000 women later migrated to the PRD region (*20*). The scale of immigration and its impact on landscape change resulting from the Qin unification of Yue should not be underestimated; this event was often compared to the Qin’s defending Hsiung-nu and the construction of the Great Wall, as noted in Pre-Han’s historical texts (*63*, *64*). During the fall of the Qin Empire, a new realm called Nanyue was established in 209 BCE, which was freed from the wars in the central China, leading to a rapid population growth (Fig. 5J). Alongside the population increase, the migrants also introduced advanced agricultural techniques, which enhanced the human ability to alter the natural environment.

The second event is the devastation of the Nanyue Realm by the Han Empire, which deployed an army of 100,000 to 200,000 sailors from 112 to 111 BCE (*20*). During this conquest, fire attacks were used, burning down the capital of Nanyue Realm, Panyu (present Guangzhou). The fire not only destroyed the city but also ran out of control to the surrounding forest. *G. pensilis* grows in swamp, which preserved stumps underwater from the fire, leaving standing *G. pensilis* stump remains with burn marks. Dating results of the *G. pensilis* stumps from the top peat layers in the PRD region show that the growth cessation times of these stumps have the average age of 2040 a B.P. (fig. S4), coinciding with the conquest time by the Han Empire. The macro-charcoal record (Fig. 7I) also shows a peak around 2.1 ka, supporting the occurrence of extensive local fire in Gaoyao, possibly related to the fire attacks during the conquest by Han Empire. After the conquest, the micro-charcoal concentration (Fig. 7J) suggests extensive consequent agriculture activities by the immigrants in this region, a byproduct of the war. Fire reconstructions from Vietnam and Maya region also indicate that early strong fire events (six times over the past 1500 years in Vietnam and burning of Witzna in 697 CE in the Maya region) are attributed to wars rather than cultivation (*65*, *66*). During the war, anthropogenic disturbances in records, including constructing canals, opening the route for supplies, and fleeing of people into the forest, have changed local hydrological conditions and land cover (*63*). Another consequence of the war was the introduction of many people with advanced cultivation technology into the PRD region, a governmental action aimed at consolidating the gains of the victory. Therefore, the disturbance to land cover caused by war is much greater than that of agricultural cultivation.

On the basis of the PCA results (Fig. 3B), *G. pensilis* is very sensitive and declines ahead of many other species due to anthropogenic disturbance. The changes in this fragile species indicate that the PRD ecosystems underwent anthropogenic alteration due to war much earlier than studies on agriculture and population suggested (*58*). After the collapse of the *G. pensilis* forest, many other forests were also destroyed by human activities, as evidenced by the variation in the AP proportion (Fig. 7F) in the coastal areas (*57*). In addition to forests, the end time of the *G. pensilis* forest marks the onset of biodiversity loss by humans in the PRD region, such as the extinction of elephants, tigers, rhinoceros, green peafowl, crocodiles, and other species (*58*, *67*–*69*). The land cover changes also affect river and estuary animals by altering terrestrial material input and the turbidity levels. The demographic history of humpback dolphin further illustrated a similar abrupt disappearance due to human development around 2.1 ka (*70*). It is a sad fact that, unlike changes caused by natural drivers, the anthropogenic-driven collapse of the peat swamp forest is difficult to reverse.

## MATERIALS AND METHODS

### Study sites and samples

The PRD is classified as a tropical and subtropical moist broadleaf forest biome (Fig. 1B) belonging to the Indo-Malayan realm (*71*). The mean annual temperature in the PRD region is c. 22°C, and annual precipitation ranges from 1600 to 1800 mm, with 80% occurring from April to September. We have studied two typical habitats that can representatively indicate the evolution history of ancient *G. pensilis* forest: One (22°55′34″ N, 112°19′45″ E, 21 m above sea level) is a depression between hills in Gaoyao; the other (23°22′33″ N, 112°42′36″ E, 6 m above sea level) is in Sihui, within a small river basin connected to the largest tributary of the Pearl River. Samples were collected from profiles exposed during the excavations of buried Chinese Swamp Cypress for material to manufacture corks by landowners. The profile in Gaoyao reached a depth of up to 6 m in its central part, while the profile in Sihui reached up to 5 m deep. The top 150 cm of the Sihui profile consisted of distinctly yellow cultivated soil, from which no samples were collected. In Gaoyao, we collected the continuous sediments from a long profile (GYS1) in the center region and selected samples only on the sedimental phase transition layers in another profile (GYS2) on the edge. In Sihui, we collected the continuous sediments from a long profile (SHS1). In both sites, we collected the wood samples from the buried huge stumps.

### Radiocarbon dating and calibration

Sediment samples were freeze-dried, and gravel, roots, and other debris were removed. About 2 to 3 g of each aliquot was treated successively with 1 M HCl, 0.2 M NaOH, and 1 M HCl. The samples were then rinsed with distilled water until neutral and subsequently freeze-dried again. The dried samples were weighed and placed into a quartz tube with silver wire and CuO. The tubes were evacuated, sealed, and combusted at 850°C to convert organic carbon into CO_2_. Afterward, the tubes were connected to a vacuum system and cracked for CO_2_ purification at the AMS-^14^C Lab in Guangzhou Institute of Geochemistry, Chinese Academy of Sciences (CAS) (*9*). A portion of purified CO_2_ was graphitized using the Zn method proposed by Xu *et al.* (*72*) and measured by accelerator mass spectrometry at the State Key Laboratory of Nuclear Physics and Technology, Peking University. To build the Bayesian age-depth model for GYS1 and SH (figs. S2 and S3), we used OxCal v4.4 (*73*) P-sequence (*74*, *75*) and a mixed curve of 50% IntCal20 and 50% SHCal20, due to the strong influence from both Northern and Southern Hemisphere air masses on the study area (*76*, *77*). The calibrated results were reported as a B.P., where 0 a B.P. = 1950 CE (*78*).

### Organic carbon and pollen analysis

TOC was calculated on the basis of the quantitative measurement of CO_2_ from oxidation at 850°C. A portion of the CO_2_ was measured for the δ^13^C value on a Finnigan MAT-251 mass spectrometer with a precision of ±0.2‰ in the State Key Laboratory of Loess and Quaternary Geology, Institute of Earth Environment, CAS. Results were reported as δ^13^C with the Pee Dee Belemnite standard. The pollen analysis was conducted in the Laboratory of Quaternary Environment, Sun Yat-sen University (*5*).

### Numerical analyses

The program R (version 4.1.1) was used for numerical analyses (*79*). PCA was conducted using the “prcomp” function in the ggbiplot package (*80*). Forty-three main palynological taxa (total percentage > 5%) and total algal spores were used in the PCA. The PCA axis 1 (PC1) explained 24.8%, the PCA axis 2 (PC2) explained a further 14.1%, and the PCA axis 3 (PC3) explained another 11.8% of the variation of the dataset. The stratigraphic palynological diagram was prepared using rioja (*81*) and vegan (*82*) packages. A constrained cluster analysis of a distance matrix was conducted, with the distance matrix of the Bray-Curtis metric and the agglomeration method of the CONISS.
