# Supplementary material for: Collapse of fragile Chinese Swamp Cypress forest
Source: Sci Adv. 2025 Apr 23;11(17):eadt1736. doi: 10.1126/sciadv.adt1736 (PMC12017334; doi:10.1126/sciadv.adt1736)
Supplement: Supplementary file 1 — Figs. S1 to S7 Tables S1 to S5 [file sciadv.adt1736_sm.pdf]

Science Advances
AAAS

Supplementary Materials for

Collapse of fragile Chinese Swamp Cypress forest

Ning Wang *et al.*

Corresponding author: Weidong Sun, weidongsun@qdio.ac.cn

Sci. Adv. **11**, eadt1736 (2025)
DOI: 10.1126/sciadv.adt1736

This PDF file includes:

Figs. S1 to S7
Tables S1 to S5

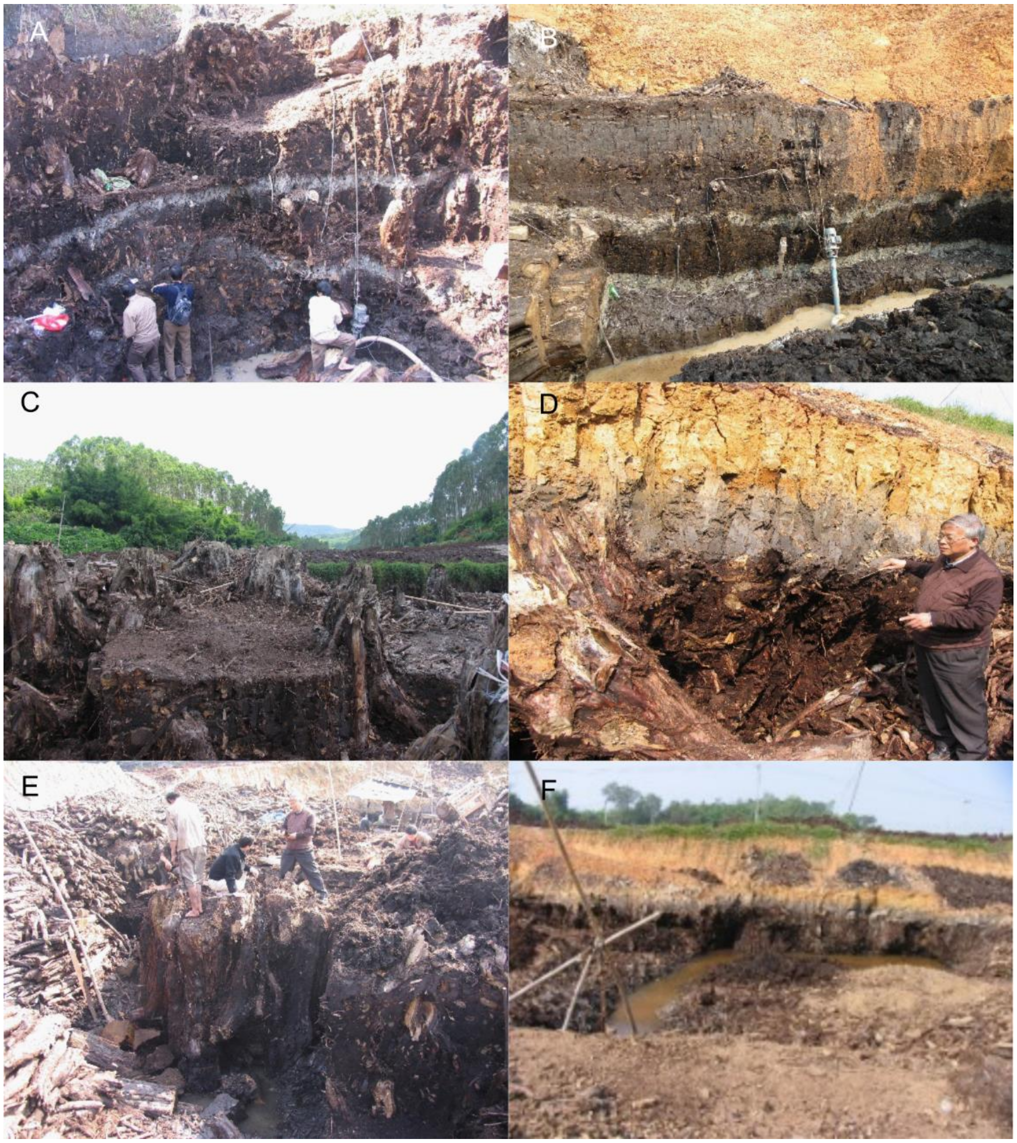

Fig. S1. Buried ancient forests in the Pearl River Delta (PRD). (A) Profile GYS1 in Gaoyao. (B) Profile GYS2 in Gaoyao. (C) Standing tree stumps in the sediments in Gaoyao. (D) Profile SHS1 in Sihui. (E) Gigantic tree stumps in SHS1. (F) Profile SHS1 submerged under the water.

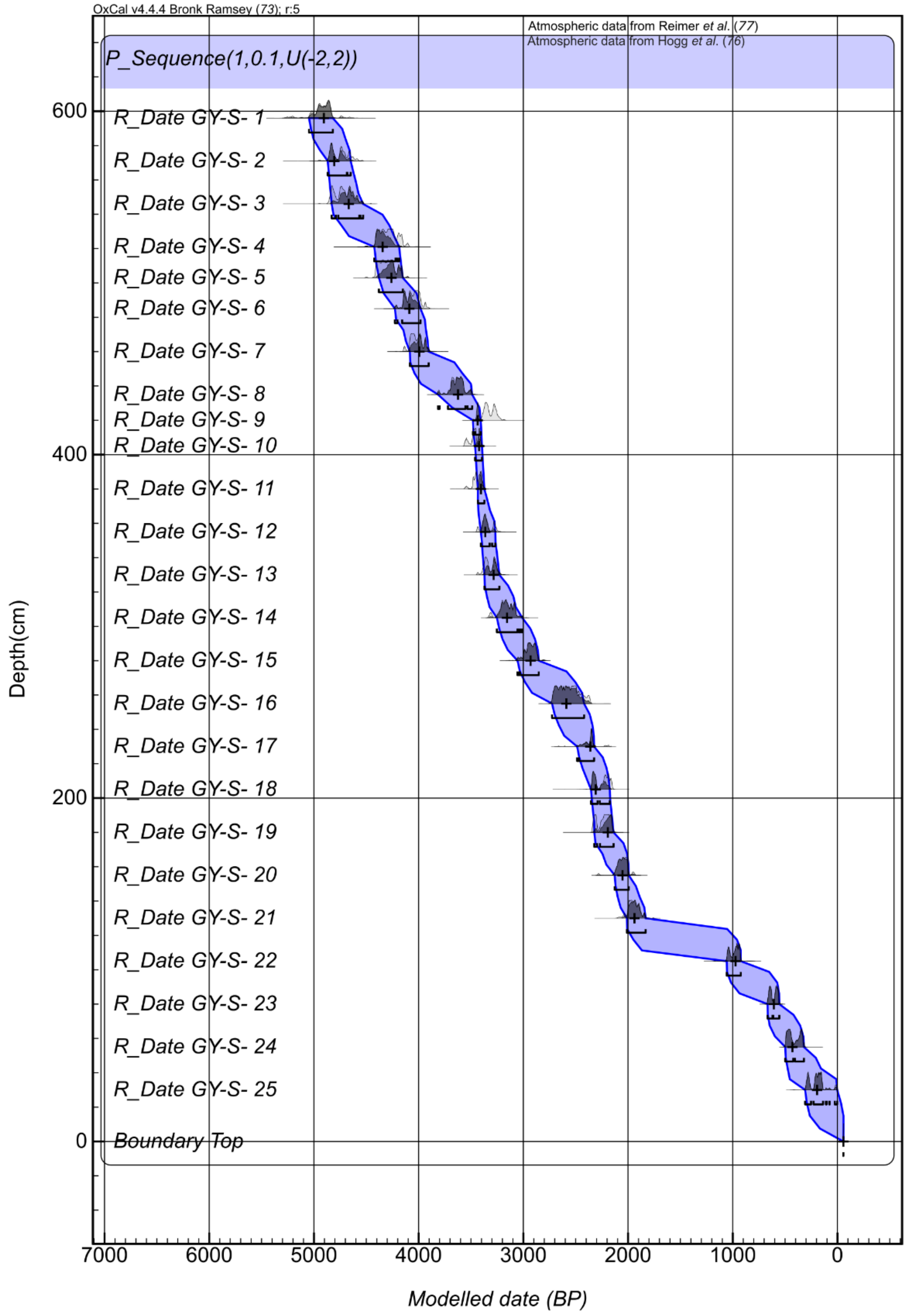

Fig. S2. Bayesian age model for GYS1 by OxCal v4.4. The model was produced in OxCal v.4.4. using a mixed curve of 50% IntCal20 and 50% SHCal20, due to the strong influence from both Northern and Southern Hemisphere air masses on the study area (*73, 76, 77*).

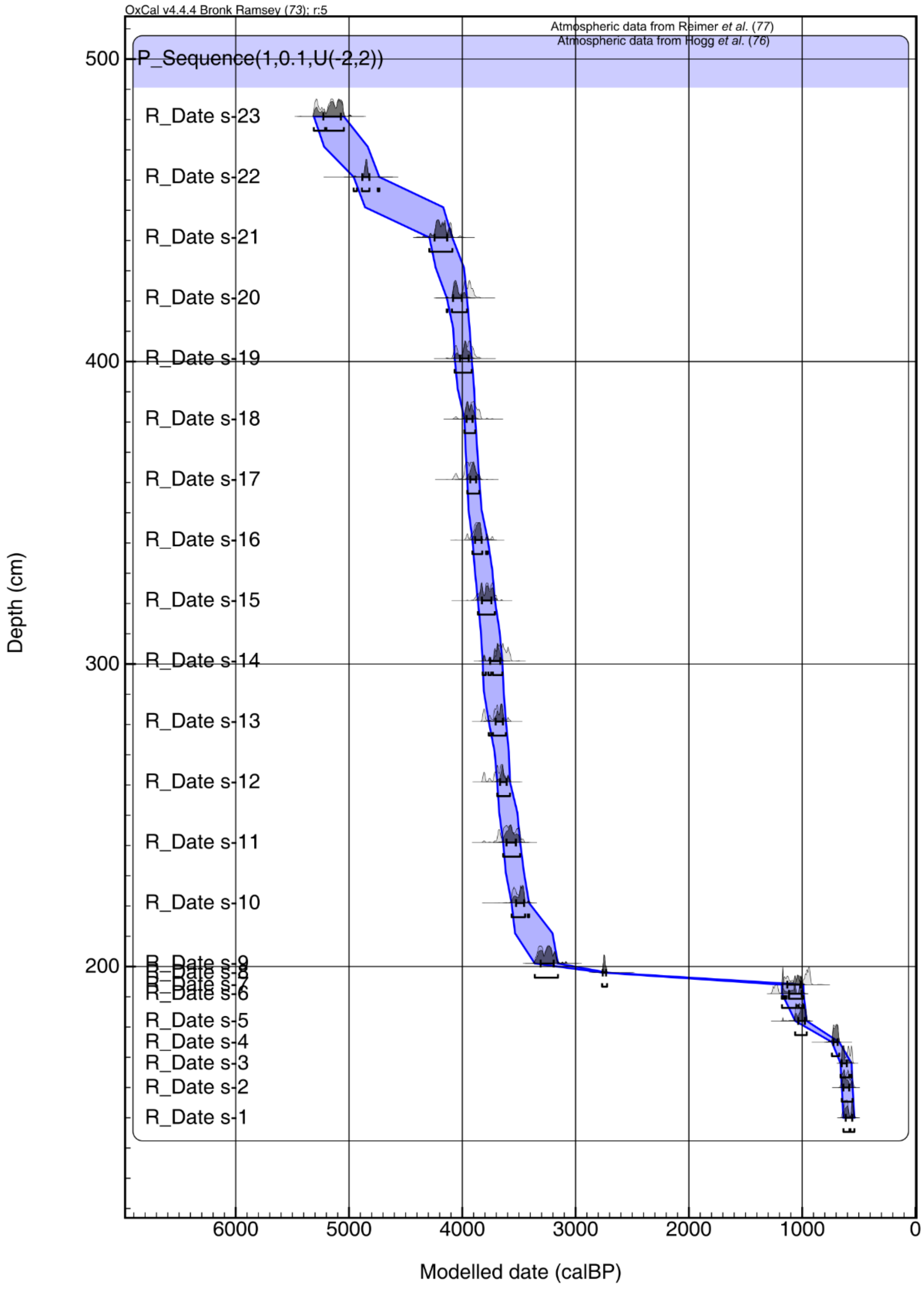

Fig. S3. Bayesian age model for SHS1 by OxCal v4.4. The model was produced in OxCal v.4.4. using a mixed curve of 50% IntCal20 and 50% SHCal20, due to the strong influence from both Northern and Southern Hemisphere air masses on the study area (*73, 76, 77*).

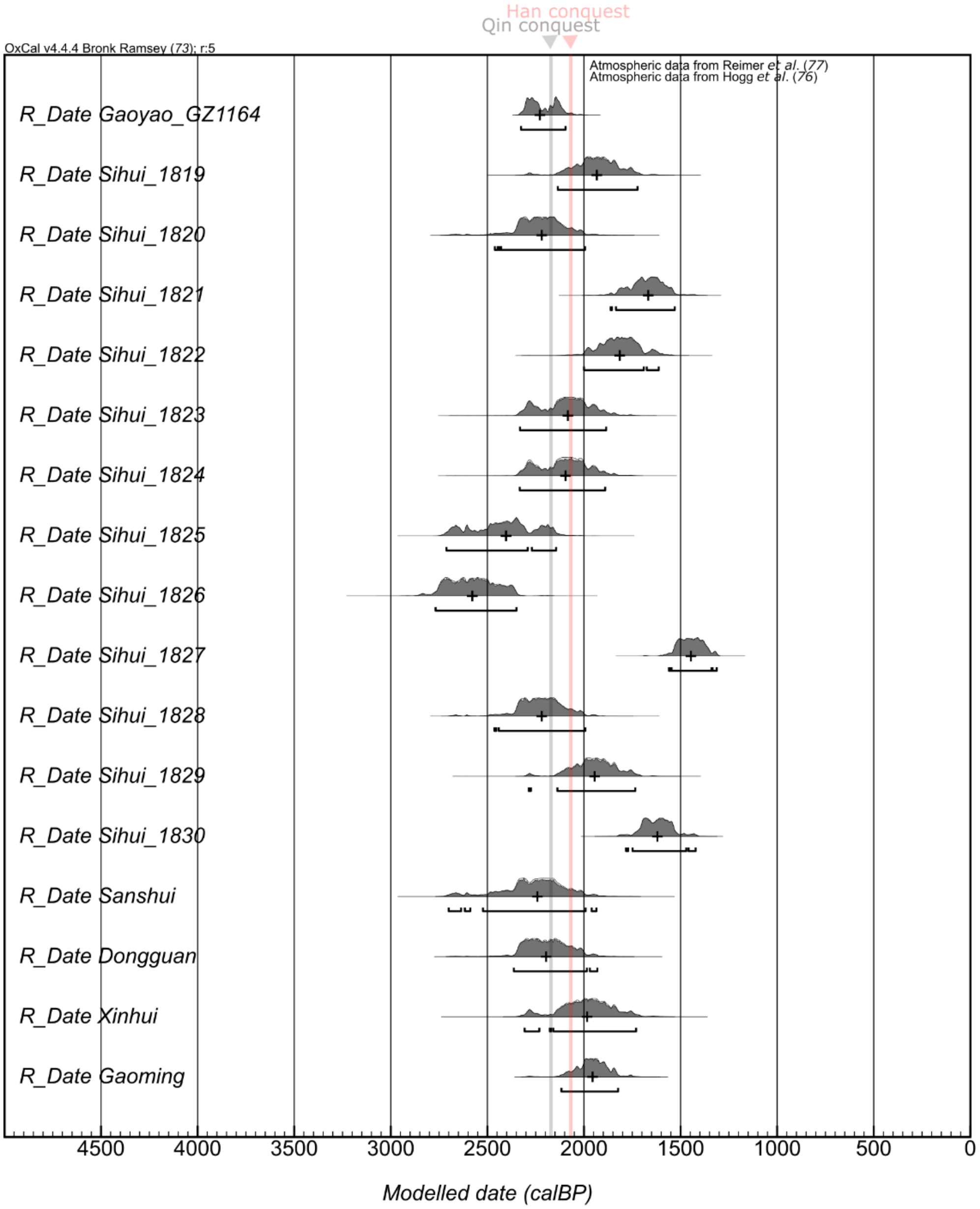

Fig. S4. Comparative age distribution of the *G. pensilis* stumps in the top peat layers in the PRD. The model was produced in OxCal v.4.4. using a mixed curve of 50% IntCal20 and 50% SHCal20, due to the strong influence from both Northern and Southern Hemisphere air masses on the study area (*73*, *76*, *77*). Data from this study and previous research, as detailed in Table S4**.**

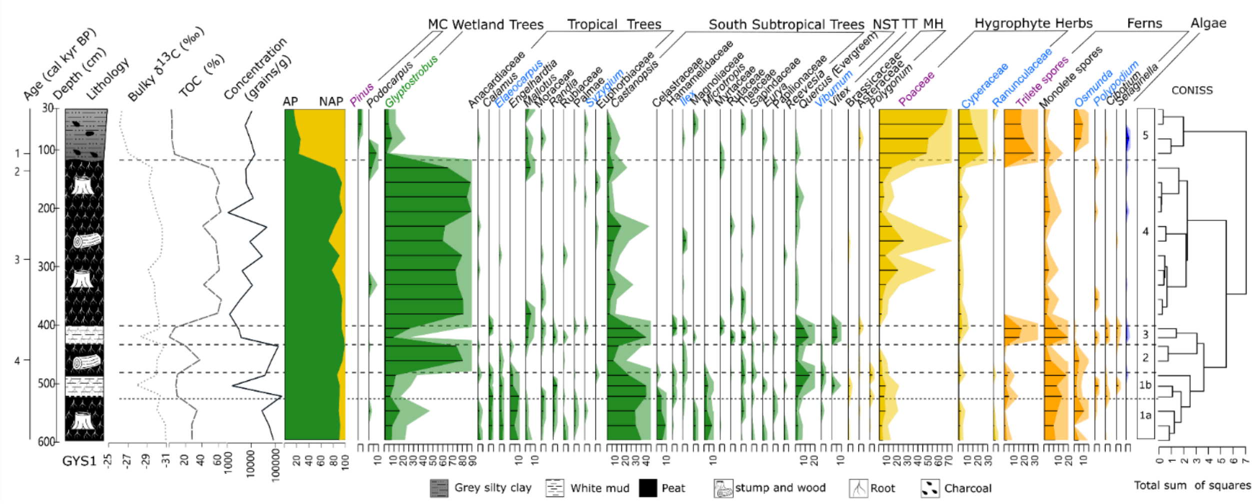

Fig. S5. Sedimentary, organic carbon and palynological records from GYS1. The main taxa are categorized into montane conifers (MC), wetland trees, tropical trees, south subtropical trees, north subtropical trees (NST), temperate trees (TT), mesophyte herbs (MH), hygrophyte herbs, ferns and total algae (details in Table S5). Trees, herbs, ferns and aquatic algae are denoted with green, yellow, orange and blue, respectively. AP denotes arboreal pollen, while NAP denotes non-arboreal pollen. Dashed lines denote the assemblage zone by CONISS (constrained incremental sum of squares).

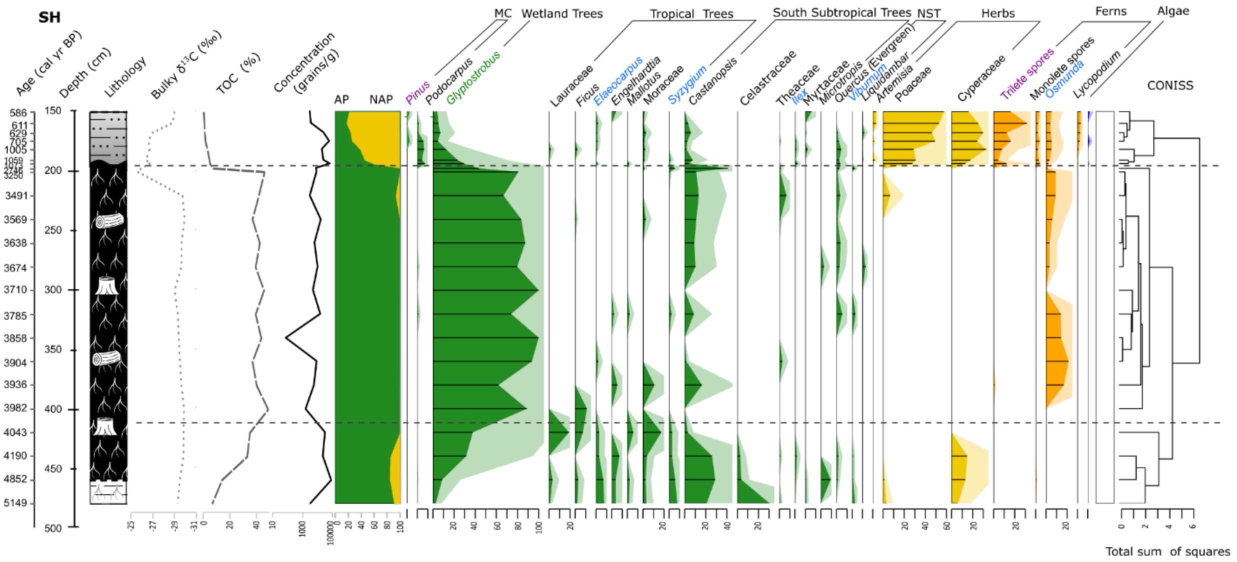

Fig. S6. Sedimentary, organic carbon and palynological records from SHS1. The main taxa are categorized into montane conifers (MC), wetland trees, tropical trees, south subtropical trees, north subtropical trees (NST), herbs, ferns and total algae (details in Table S5). Trees, herbs, ferns and aquatic algae are denoted with green, yellow, orange and blue color. AP denotes arboreal pollen, while NAP denotes non-arboreal pollen. Dash lines denote the assemblage zone by CONISS (constrained incremental sum of squares).

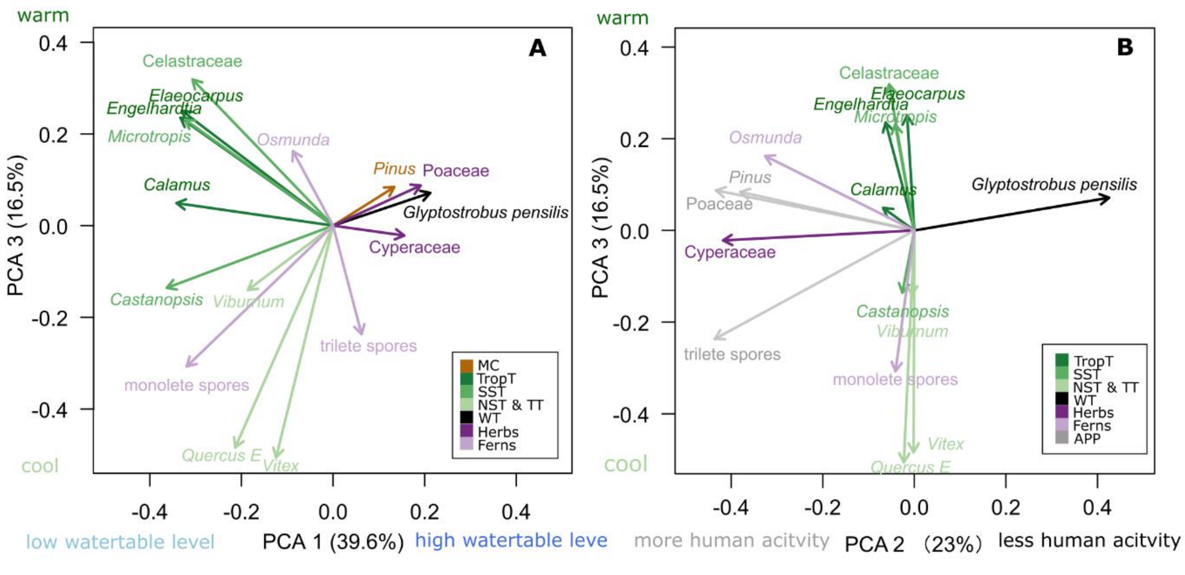

Fig. S7.PCA ordination diagram of selected palynological taxa (16 species) from GYS1. (A) PCA 1 versus PCA 3. (B) PCA 2 versus PCA 3. Different colors denote montane conifers (MC), tropical trees (TropT), south subtropical trees (SST), north subtropical trees (NST), temperate trees (TT), wetland trees (WT), anthropogenic and pioneer plants (APP), herbs, and ferns.

Location	Altitude (m)	Latitude (N)	Longitude (E)	Annual temperature (℃)	Mean temperature in Jan. (℃)	Mean temperature in Jul.(℃)	Annual precipitation (mm)
Doumen, Guangdong, China	32	22°22'	113°12'	22.0	18.3	26.5	2231
Guangzhou, Guangdong, China	69	23°11'	113°22'	21.6	12.8	28.5	1720
Huazhou, Guangdong, China	32	21°37'	110°39'	22.6	14.7	28.6	1800
Pingyuan, Guangdong, China	167	24°22'	115°54'	20.6	11	28.5	1630
Qujiang, Guangdong, China	135	24°39'	113°38'	20.1	9.6	28.9	1640
Chenzhou,Hunan, China	355-678	26°12'	113°31'	17.6	5.8	28.8	1469
Qianshan, Jiangxi, China	84-104	28°12'	117°32'	17.8	5.6	29.4	1920
Nanchang, Jiangxi , China	54-59	28°46'	115°49'	17.7	6.5	32.0	1520
Yiyang, Jiangxi, China	42-88	28°22'	117°24'	18.0	5.4	29.1	1816
Yujiang, Jiangxi, China	46	28°17'	116°58'	18.2	5.2	29.3	1725
Guilin, Guangxi, China	183	25°05'	110°18'	19.3	8.3	28.5	1780
Hepu, Guangxi, China	69	21°39'	109°13'	22.4	15.2	28.8	1650
Zhangping, Fujian, China	768-779	25°03'	117°18'	19.0	3.7	27.4	1796
Yongchun, Fujian, China	778-873	25°31'	118°06'	18.3	4.9	29.8	1800
Jian'ou, Fujian, China	409-575	27°01'	118°36'	19.3	9.6	28.5	1696
Pingnan, Fujian, China	1280	27°01'	118°52'	15.3	5.2	26.3	2090
Shaowu, Fujian, China	584	27°31'	117°27'	17.7	4.3	29.6	1802
Pucheng, Fujian, China	356	27°38'	118°18'	17.4	3.9	31.2	1782
Funing, Yunnan, China	370	23°49'	105°55'	19.3	10.8	25.3	1200
Kunming, Yunnan, China	1980	25°09'	102°44'	15.2	7.8	19.9	1400
Jigongslian, Henan, China	NA	31°46'	114°13'	12.1	NA	NA	1347
Hangzhou, Zhejiang, China	26	30°15'	120°06'	16.1	3.6	28.8	1401
Dak Lak, Vietnam	570	13°14'	108°11'	24.6	21.7	NA	~1550
Borikhamxai, Laos	615	18°02'	105°03'	23.8	19.2	NA	~2000

Table S1. Climate conditions of the extant native *G. pensilis* habitats. This table lists the altitude, latitude, longitude, annual temperature, mean temperature in January and July, annual precipitation of extant native *G. pensilis* habitats (*8*, *12*).

Lab NO.	Field NO.	Depth (cm)	^{14}C age (yr BP ± 1σ)	Modeled Calendar age[a] (yr BP)	Modeled[b] mean age (yr BP ± 1σ)	$\delta^{13}C$ (‰)	TOC (%)
Bulk organic carbon in GYS1							
GZ1126	GY-S- 1	596	4341 ± 62	5047 - 4820	4915 ± 70	-30.9	28.4
GZ1134	GY-S- 2	571	4249 ± 51	4868 - 4649	4782 ± 62	-30.8	28.1
GZ1135	GY-S- 3	546	4227 ± 54	4831 - 4531	4673 ± 68	-30.0	34.6
GZ1136	GY-S- 4	521	3885 ± 49	4423 - 4186	4336 ± 59	-30.3	10.1
GZ1137	GY-S- 5	503	3892 ± 41	4381 - 4151	4259 ± 63	-27.8	8.8
GZ1138	GY-S- 6	485	3730 ± 40	4227 - 3982	4091 ± 55	-29.9	10.5
GZ1139	GY-S- 7	460	3700 ± 31	4084 - 3905	3995 ± 50	-30.0	38.3
GZ1140	GY-S- 8	435	3412 ± 38	3816 - 3489	3626 ± 61	-30.7	20.3
GZ1141	GY-S- 9	420	3136 ± 38	3482 - 3407	3437 ± 15	-28.2	1.0
GZ1142	GY-S- 10	405	3280 ± 29	3462 - 3396	3425 ± 15	-30.2	7.5
GZ1143	GY-S- 11	380	3256 ± 31	3434 - 3373	3405 ± 14	-30.1	59.6
GZ1144	GY-S- 12	355	3155 ± 33	3405 - 3268	3357 ± 30	-29.8	64.0
GZ1145	GY-S- 13	330	3138 ± 34	3372 - 3229	3294 ± 41	-29.7	43.0
GZ1146	GY-S- 14	305	3004 ± 34	3253 - 3009	3151 ± 56	-29.0	61.3
GZ1152	GY-S- 15	280	2849 ± 33	3057 - 2852	2934 ± 49	-30.3	59.5
GZ1153	GY-S- 16	255	2499 ± 43	2727 - 2421	2584 ± 87	-30.5	57.0
GZ1154	GY-S- 17	230	2364 ± 34	2486 - 2325	2379 ± 46	-30.5	41.9
GZ1155	GY-S- 18	205	2302 ± 41	2354 - 2175	2283 ± 54	-29.9	63.6
GZ1156	GY-S- 19	180	2267 ± 34	2323 - 2140	2202 ± 44	-29.6	57.4
GZ1157	GY-S- 20	155	2107 ± 34	2129 - 1993	2054 ± 38	-29.1	61.3
GZ1158	GY-S- 21	130	2025 ± 41	2011 - 1833	1936 ± 46	-29.5	53.1
GZ1159	GY-S- 22	105	1091 ± 38	1058 - 924	983 ± 44	-27.0	7.0
GZ1160	GY-S- 23	80	672 ± 33	668 - 556	611 ± 34	-26.6	3.7
GZ1161	GY-S- 24	55	388 ± 33	499 - 320	414 ± 57	-26.1	4.0
GZ1162	GY-S- 25	30	217 ± 34	309 - 3	200 ± 65	-26.3	3.3
Bulk organic carbon in GYS2							
GZ1787	GY2-S- 3	400	3180 ± 35	3453 - 3262	3382 ± 45	-27.1	3.5
GZ1788	GY2-S- 4	420	3285 ± 34	3566 - 3400	3484 ± 46	-27.3	3.8
GZ1789	GY2-S- 5	490	3847 ± 30	4402 - 4097	4231 ± 68	-27.3	3.7
GZ1790	GY2-S- 6	525	3960 ± 32	4517 - 4253	4383 ± 66	-27.3	4.0
Stump in GYS1[c]							
GZ1163	GY-T-1I		2433 ± 34	2699 - 2347	2476 ± 101	-26.7	
GZ1164	GY-T-1O		2207 ± 36	2326 - 2095	2211 ± 71	-28.4	
GZ1165	GY-T-2O		3748 ± 34	4228 - 3976	4075 ± 63	-26.3	
GZ1167	GY-T-3O		3945 ± 36	4511 - 4244	4357 ± 64	-26.1	
GZ1240	GY-T-3I		4314 ± 36	4965 - 4825	4870 ± 45	-24.7	

Table S2. Radiocarbon and organic carbon results of stumps and sediments from GYS1 and GYS2 in Gaoyao. Bulk organic carbon in GYS1 and GYS2 is the result of bulky organic matter in sediments, and stump in GYS1 is the result of wood sample.

[a] The calendar ages are reported with a 95.45% confidence interval.

[b] The age model methodology is detailed in the "Radiocarbon dating and calibration" section of Materials and Methods.
[c] Stump sample field numbers with the suffix "I" denote the innermost portions, while those with "O" indicate the outermost portions of the stumps.

Lab NO.	Field NO.	Depth (cm)	^{14}C age (yr BP ± 1σ)	Modeled Calendar age[a] (yr BP)	Modeled[b] mean age (yr BP ± 1σ)	δ^{13}C (‰)	TOC (%)
Bulk organic carbon in SHS1							
GZ963	s-1	150	617 ± 30	636 - 539	586 ± 29	-29.0	0.8
GZ962	s-2	160	642 ± 34	650 - 555	611 ± 27	-28.8	1.1
GZ961	s-3	168	651 ± 27	662 - 564	629 ± 23	-27.0	1.6
GZ960	s-4	175	822 ± 26	737 - 673	705 ± 19	-26.6	1.8
GZ958	s-5	182	1148 ± 27	1062 - 960	1005 ± 29	-26.7	3.4
GZ957	s-6	191	1250 ± 29	1178 - 987	1059 ± 57	-26.4	5.0
GZ956	s-7	194	1083 ± 35	1180 - 990	1073 ± 59	-26.8	5.5
GZ955	s-8	198	2634 ± 26	2766 - 2723	2746 ± 15	-26.0	7.9
GZ773	s-9	201	3068 ± 35	3360 - 3156	3250 ± 58	-25.6	46.3
GZ774	s-10	221	3305 ± 29	3565 - 3411	3491 ± 35	-29.7	41.9
GZ775	s-11	241	3375 ± 45	3640 - 3489	3569 ± 41	-29.9	37.2
GZ776	s-12	261	3448 ± 27	3692 - 3579	3638 ± 29	-29.6	42.8
GZ777	s-13	281	3456 ± 28	3767 - 3615	3674 ± 32	-29.6	39.4
GZ778	s-14	301	3429 ± 29	3821 - 3647	3710 ± 44	-29.0	45.8
GZ779	s-15	321	3537 ± 30	3863 - 3712	3785 ± 42	-29.3	39.4
GZ780	s-16	341	3587 ± 27	3910 - 3775	3858 ± 29	-29.4	43.7
GZ781	s-17	361	3642 ± 30	3956 - 3851	3904 ± 26	-29.5	37.4
GZ782	s-18	381	3624 ± 29	3981 - 3886	3936 ± 27	-29.6	40.4
GZ783	s-19	401	3665 ± 30	4067 - 3917	3982 ± 39	-29.9	49.0
GZ784	s-20	421	3673 ± 30	4137 - 3959	4043 ± 39	-29.8	35.1
GZ785	s-21	441	3813 ± 31	4291 - 4089	4190 ± 56	-29.8	33.4
GZ786	s-22	461	4302 ± 30	4959 - 4733	4852 ± 31	-29.5	14.5
GZ787	s-23	481	4544 ± 33	5311 - 5046	5149 ± 77	-29.3	6.9
Stump in SHS1[c]							
GZ585	T-1O		3232 ± 27	3477 - 3373	3422 ± 25		
GZ586	T-1I		3893 ± 28	4413 - 4159	4310 ± 62		
GZ588	T-2O		3406 ± 23	3692 - 3569	3626 ± 39		
GZ589	T-2I		3661 ± 25	4083 - 3880	3965 ± 54		
GZ943	T-3O		3245 ± 31	3486 - 3374	3433 ± 33		
GZ944	T-3I		3569 ± 36	3969 - 3716	3834 ± 64		
GZ948	T-4O		2892 ± 34	3144 - 2877	3001 ± 60		
GZ949	T-4I		3820 ± 40	4400 - 4004	4192 ± 76		

Table S3. Radiocarbon and organic carbon results of stumps and sediments from SHS1 in Sihui. Bulk organic carbon in SHS1 is the result of bulky organic matter in sediments, and stump in SHS1 is the result of wood sample.

[a] The calendar ages are reported with a 95.45% confidence interval.

[b] The age model methodology is detailed in the “Radiocarbon dating and calibration” section of Materials and Methods.

[c] Stump sample field numbers with the suffix “I” denote the innermost portions, while those with “O” indicate the outermost portions of the stumps.

location	Lab. NO.	14C age (yr BP± 1σ)			Calendar age[a] (cal yr BP)			Mean calibrated age (cal yr BP± 1σ)			ref. [b]
Gaoyao	GZ1164	2207	±	36	2326	-	2095	2211	±	71	This study[b]
Sihui	GZ948	2892	±	34	3142	-	2878	3001	±	60	This study
Sihui	1819	2010	±	85	2135	-	1722	1935	±	110	Ref. 18
Sihui	1820	2250	±	90	2465	-	1995	2221	±	117	Ref. 18
Sihui	1821	1790	±	70	1865	-	1530	1673	±	83	Ref. 18
Sihui	1822	1910	±	80	2001	-	1611	1815	±	97	Ref. 18
Sihui	1823	2130	±	90	2331	-	1885	2094	±	123	Ref. 18
Sihui	1824	2140	±	90	2333	-	1891	2106	±	122	Ref. 18
Sihui	1825	2370	±	90	2711	-	2145	2413	±	158	Ref. 18
Sihui	1826	2540	±	95	2769	-	2350	2576	±	121	Ref. 18
Sihui	1827	1580	±	60	1560	-	1313	1447	±	62	Ref. 18
Sihui	1828	2250	±	90	2465	-	1995	2221	±	117	Ref. 18
Sihui	1829	2020	±	85	2287	-	1732	1948	±	111	Ref. 18
Sihui	1830	1740	±	65	1785	-	1421	1621	±	76	Ref. 18
Sanshui	KWG15	2270	±	110	2698	-	1939	2256	±	158	Ref. 7
Dongguan	KWG40	2220	±	90	2362	-	1933	2191	±	113	Ref. 7
Xinhui	GC462	2050	±	100	2306	-	1729	1990	±	133	Ref. 7
Gaoming	KWG1722	2030	±	60	2118	-	1822	1956	±	78	Ref. 7

Table S4. Distribution of terminal ages for buried *G. Pensilis* stumps in PRD. This table lists the compiled results of the stump samples in the PRD region.

[a] The calendar ages are reported with a 95.45% confidence interval.

[b] The data in this study pertain solely to the outmost parts of stumps, reflecting the time when the tree died. Data from other references may not specify this but are typically employed to denote the ending time of ancient forests in respective studies.

**Ecological groups**	**Pollen taxa**
**Wetland trees**	*Glyptostrobus pensilis*
**Montane conifer**	*Pinus, Podocarpus*
**Tropical trees**	Anacardiaceae*, Calamus, Elaeocarpus, Engelhardtia, Mallotus,* Moraceae, *Randia,* Rubiaceae*,* Euphorbiaceae*, Syzygium*, Lauraceae*, Ficus, Aporosa,* Palmae, Melastomataceae Apocynaceae, *Iodes*
**South subtropical trees**	*Castanopsis,* Celastraceae, Hamamelidaceae, *Ilex,* Magnoliaceae, *Microtropis,* Myrtaceae, Rutaceae, Theaceae, Sapindaceae, *Eurya,* Papilionaceae, *Reevesia,* Araliaceae, Myrsinaceae, *Camellia, Nyssa, Distylium, Symplocos*
**North subtropical trees**	*Quercus* (evergreen), *Liquidambar, Viburnum*
**Temperate trees**	*Vitex*
**moderate herbs**	Asteraceae, Brassicaceae, *Polygonum,* Loranthaceae, Thymelaeaceae, *Artemisia*
**Hygrophyte herbs**	Poaceae, Cyperaceae, Ranunculaceae, Liliaceae
**Fern**	trilete spores, Osmunda, monolete spores, *Polypodium, Cibotium, Selaginella*
**Aquatic algae**	*Zygnema, Potamogeton, Concentricystes, Typha, Mougeotia*

Table S5. Ecological groupings of palynological taxa. The ecological groupings of the main taxa from GYS1 and SHS1 profiles.
